# Spatiotemporal Evolution of Interfacial pH and H_2_ Level During Degradation of Mg-Al-Y-Zn-Gd Alloys in Hanks’ Balanced Salt Solution

**DOI:** 10.3390/molecules31183359

**Published:** 2026-09-21

**Authors:** Cheng Wang, Ming Yin, Ziwei Yuan, Tingting Zhang, Ziyue Xu, Yuxuan Li, Man Xu, Juichi Chang, Yi Shao, Chao Sun, Dali Wei, Qiangsheng Dong, Chenglin Chu, Feng Xue, Huan Liu, Jing Bai

**Affiliations:** 1 School of Materials Science and Engineering, Southeast University, Nanjing 211189, China; 2 College of Materials Science and Engineering, Hohai University, Changzhou 213200, China; 3 Institute of Biomedical Devices (Suzhou), Southeast University, Suzhou 215163, China; 4 School of Materials Science and Engineering, Nanjing Institute of Technology, Nanjing 211167, China

**Keywords:** local pH, local H_2_ level, Mg alloy, Hanks’ balanced salt solution (HBSS)

## Abstract

Understanding the dynamic interfacial microenvironment of degrading Mg alloys is critical for elucidating the evolution of localized corrosion under physiological conditions. Here, spatially resolved microsensors were used to characterize the local pH and H_2_ concentration at the interfaces of Mg-Y-Al-Zn alloys without Gd (0Gd) and with 3 wt.% Gd (3Gd) during degradation in Hanks’ balanced salt solution (HBSS). Both local pH and H_2_ concentration rapidly increased at the interfaces of 0Gd and 3Gd immediately after immersion in HBSS, accompanied by pronounced near-surface pH and H_2_ gradients. The initial local pH and H_2_ level for 0Gd were comparable to or slightly lower than those of 3Gd. However, 0Gd subsequently exhibited distinct localized high pH and H_2_ level associated with the filiform corrosion. From 3 to 12 h, the localized pH and H_2_ features of 0Gd progressively intensified, while 3Gd maintained a relatively weaker and more spatially distributed response. During prolonged immersion from 16 to 18 h, localized H_2_ accumulation also emerged on 3Gd despite comparatively moderate local alkalization. These findings reveal the dynamic and spatially heterogeneous nature of the Mg corrosion microenvironment and demonstrate that simultaneous mapping of local pH and H_2_ provides complementary information for resolving its spatiotemporal evolution.

## 1. Introduction

Magnesium (Mg) and its alloys have attracted considerable attention as biodegradable metals, which are expected to provide temporary mechanical support while gradually degrading [1,2,3,4,5,6,7]. However, the relatively high electrochemical activity of Mg in aqueous environments remains a major challenge for its biomedical application. Rapid localized degradation can lead to premature loss of mechanical integrity, while the accompanying generation of hydrogen and alkalization of the surrounding environment may further influence the biological response and degradation behavior of the implant [8,9,10,11,12,13,14,15]. Therefore, achieving a predictable and spatially controlled degradation behavior requires a detailed understanding of the interfacial processes governing Mg corrosion under physiological conditions.

The degradation of Mg in aqueous and physiological environments is governed by coupled anodic and cathodic reactions. The anodic dissolution of Mg substrate generates Mg^2+^ ions, while cathodic reduction of water and oxygen produces H_2_ and OH^−^ [16,17,18,19,20,21]. The generation of OH^−^ can increase the pH adjacent to the Mg surface, while the resulting alkaline environment promotes the precipitation and transformation of Mg-containing corrosion products [22,23,24,25,26,27,28]. Importantly, these reactions occur within a thin interfacial region where the transport of reactive species, buffering components, dissolved metal ions, and corrosion products differs substantially from that in the bulk electrolyte [29,30,31,32]. Previous studies using localized electrochemical measurements have demonstrated pronounced near-surface alkalization of Mg alloys in simulated physiological media, even when the bulk electrolyte is buffered [33,34,35,36,37,38,39,40]. Local pH is also closely coupled with hydrogen evolution observed preferentially at active localized corrosion sites. Local H_2_ concentration is attributed to the cathodic hydrogen evolution, whereas local pH reflects the net consequence of OH^−^ generation, transport, buffering, and interfacial chemical reactions [29,41,42,43,44,45,46,47]. Following the evolution of local pH and H_2_ concentration from the initial corrosion stage to the development of localized corrosion sites may help clarify how localized corrosive activity is established, intensified, and redistributed at the Mg/electrolyte interface. Therefore, the evolution of local pH and H_2_ concentration could be considered as spatially resolved indicators of the electrochemical activity developing at the degrading interface of Mg alloys.

In this work, spatially resolved local pH and H_2_ measurements were employed to investigate the spatiotemporal evolution of the interfacial corrosion microenvironment of Mg-Al-Y-Zn alloys with and without 3 wt.% Gd (denoted as 0Gd and 3Gd, respectively) during degradation in HBSS. Alloying Gd has been reported to influence the corrosion behavior of Mg alloys through changes in grain size, second-phase formation and distribution, and micro-galvanic interactions [48,49,50,51,52]. These comprehensive corrosion behaviors of the alloys present in this work have been previously studied in a NaCl electrolyte [53]. Thus, both local pH and H_2_ concentration adjacent to the degrading Mg surface were simultaneously monitored in the vertical and horizontal directions. The evolution of local pH and H_2_ was further tracked from the initial stage to prolonged immersion to visualize the development, localization, and redistribution of corrosion activity. Particular attention was given to the differences between 0Gd and 3Gd in the formation of local alkaline and H_2_-rich regions, and to the relationship between local H_2_ evolution and alkalization. This study aims to demonstrate the value of simultaneous local pH and H_2_ mapping for understanding the heterogeneous degradation behavior of Mg alloys in physiological environments.

## 2. Results and Discussions

**Figure 1:**
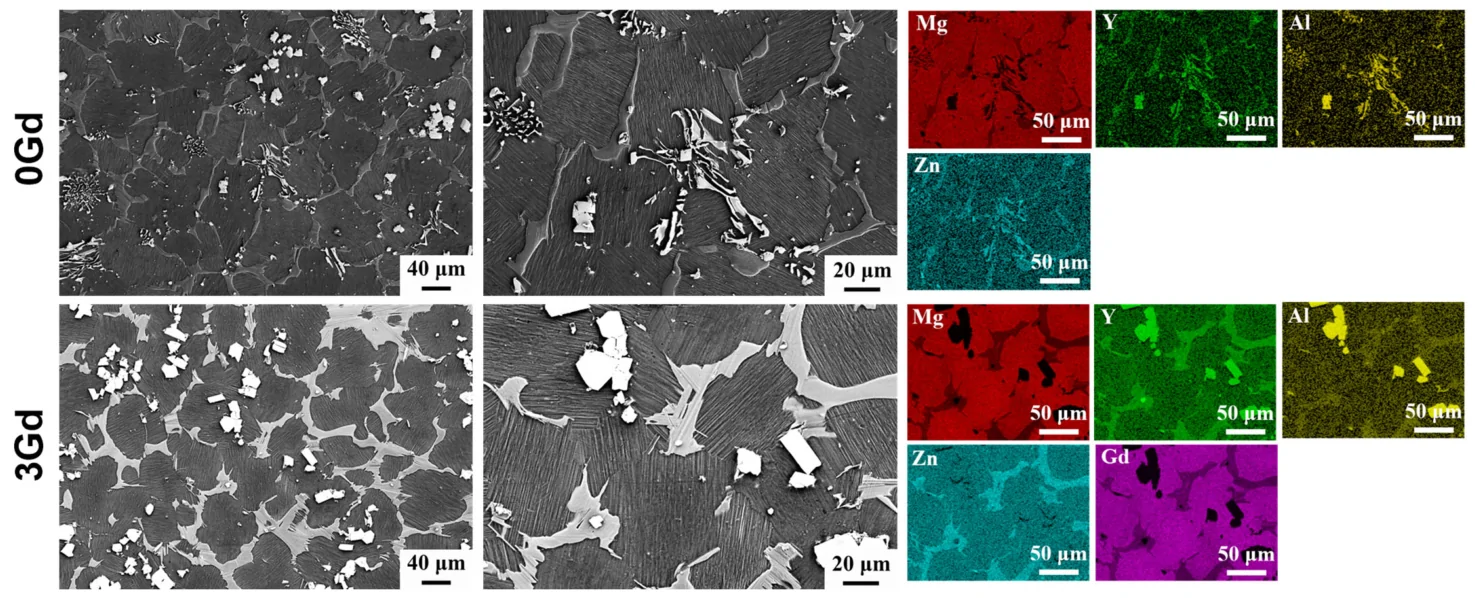
The SEM image and EDS analysis of 0Gd and 3Gd.

The microstructural characteristics and elemental distributions of the 0Gd and 3Gd alloys were first examined by SEM and EDS, as shown in Figure 1. Both alloys exhibited an α-Mg matrix with secondary-phase or element-enriched regions distributed within the microstructure. Compared with 0Gd, the incorporation of Gd into the 3Gd alloy resulted in an evident modification of the morphology and spatial distribution of the secondary/enriched regions.

The EDS maps further confirmed the presence and distribution of Gd in the 3Gd alloy, indicating that Gd addition altered not only the nominal chemical composition but also the local microstructural characteristics of the alloy. These microstructural modifications provide a possible basis for the different spatial corrosion responses observed between 0Gd and 3Gd. Such microstructural differences are expected to influence the local electrochemical activity of the Mg surface during degradation. In particular, variations in phase distribution and local chemical composition may generate spatially different anodic and cathodic activities and consequently lead to heterogeneous corrosion. Our previous studies have demonstrated that Gd addition can substantially modify the second-phase constitution and distribution and consequently alter the corrosion behavior of Mg alloys [53].

**Figure 2:**
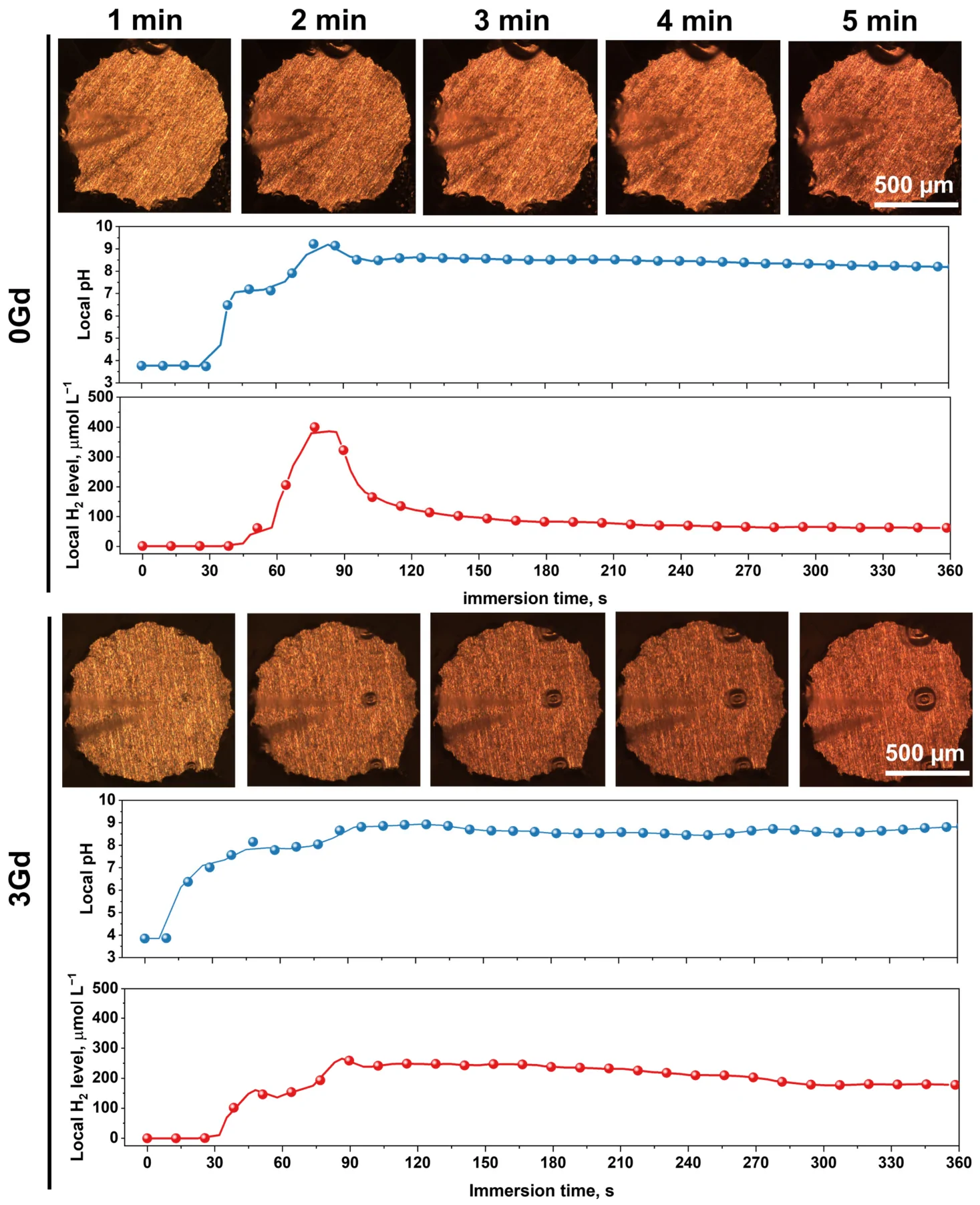
The immediate evolution of local pH and H_2_ level above the surface center of 0Gd and 3Gd exposed to HBSS during the first 5 min. The microsensors were initially positioned 100 µm above sample surface in air, and both the samples and microsensors were brought into contact with HBSS at approximately 30 s after the start of recording.

The immediate evolution of local pH and H_2_ above the center of the alloy surfaces during the first 5 min of immersion is presented in Figure 2. Both 0Gd and 3Gd exhibited rapid changes in the local chemical environment immediately after exposure to HBSS. For 0Gd, the local pH increased rapidly from the initial solution condition, accompanied by a pronounced increase in the local H_2_ concentration. After reaching a maximum or quasi-steady level, both signals gradually approached a relatively stable state. A similar response was observed for 3Gd, although the magnitude and temporal evolution of the local pH and H_2_ signals were substantially different from those of 0Gd. The simultaneous increase in local alkalinity and H_2_ concentration is consistent with the characteristic cathodic reactions occurring during Mg degradation. Consequently, the generation of H_2_ and OH^−^ provides a direct chemical signature of cathodic activity at the Mg/electrolyte interface. The temporal correlation between the increase in H_2_ concentration and local pH therefore demonstrates that corrosion initiation is accompanied by the rapid establishment of a chemically distinct interfacial environment. Importantly, 3Gd exhibited a substantially attenuated local response compared with 0Gd, particularly in terms of H_2_ accumulation and local alkalization. At this stage, no obvious localized corrosion was observed on either alloy surface, suggesting that the difference mainly reflects the distinct early stage interfacial corrosion activity of the two alloys before pronounced localized corrosion develops.

**Figure 3:**
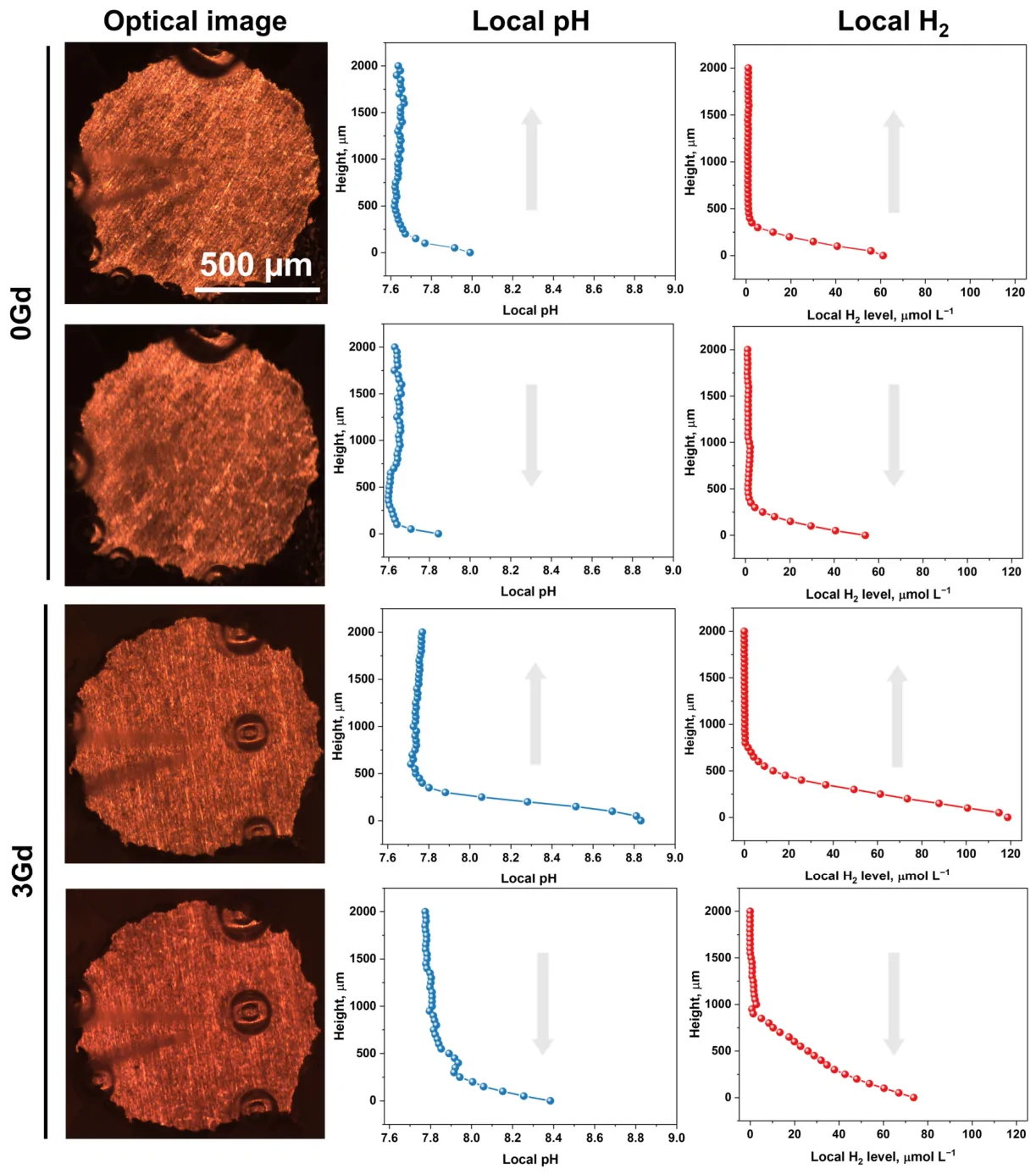
The profiles of local pH and H_2_ level above the surface center of 0Gd and 3Gd exposed to HBSS. The grey arrows indicate the scanning direction of the microsensors from surface to bulk or the opposite. The zero point on the Y-axis corresponds to a probe-to-surface distance of 100 μm.

Although the temporal measurements in Figure 2 demonstrate rapid changes in local pH and H_2_, they do not by themselves establish whether these changes are restricted to the vicinity of the Mg surface or represent a more general change in the bulk electrolyte. To clarify this point, vertical profiles of local pH and H_2_ were obtained by moving the microsensors between the alloy surface and the bulk HBSS in both scanning directions, as shown in Figure 3. A pronounced spatial gradient was observed for both parameters. Close to the alloy surface, both local pH and H_2_ concentrations deviated strongly from the bulk values. Although the profiles obtained from the two scanning directions were not identical, both exhibited the same characteristic near-surface-to-bulk trend. The differences between the two scans, particularly in the vicinity of the alloy surface, can be attributed to the transient evolution of the corrosion-induced interfacial gradients during the measurement.

These results provide direct evidence that the corrosion-induced chemical perturbation is strongly confined to the near-surface region. Mg degradation continuously generates a localized chemical microenvironment in which reaction products and cathodic products accumulate before being transported into the bulk solution. The vertical profiles also highlight the importance of spatially resolved measurements for understanding Mg degradation. A conventional measurement performed in the bulk electrolyte would largely average out these near-surface gradients and could therefore miss the pronounced chemical heterogeneity generated directly at the degrading interface. The local pH and H_2_ measurements consequently provide complementary information about the reaction zone immediately adjacent to the Mg surface. Interestingly, before the development of obvious localized corrosion features, both local pH and H_2_ level for 0Gd were slightly lower than those of 3Gd. This trend was also consistent with the initial time-dependent measurements shown in Figure 2.

**Figure 4:**
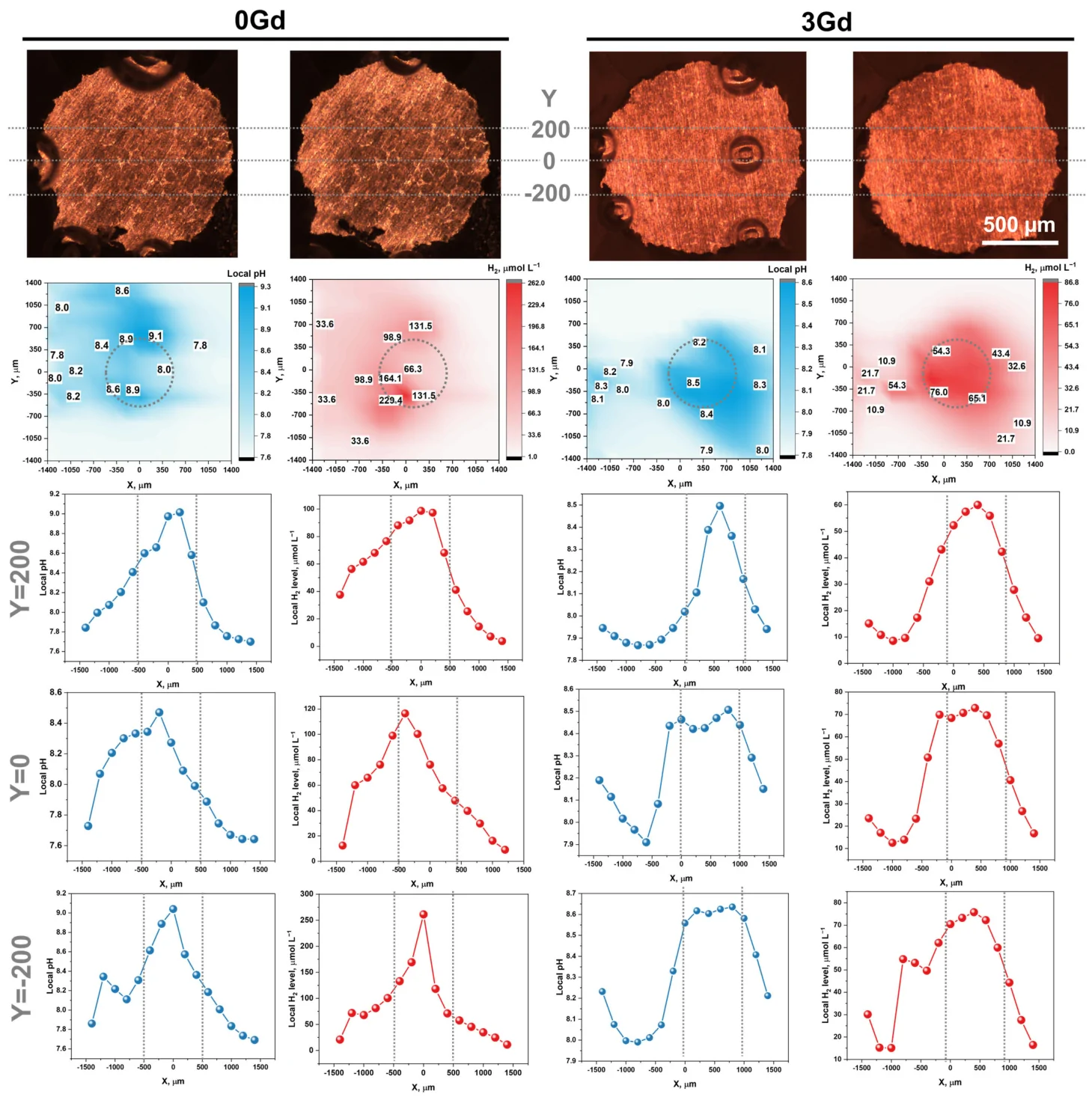
The horizontal distribution of local pH and H_2_ level at above the surface of 0Gd and 3Gd exposed to HBSS by 2 h. The grey circles and dash lines indicate the sample area.

To further resolve the lateral distribution of corrosion activity, horizontal mapping of local pH and H_2_ was performed over the alloy surfaces after 2 h of immersion, with representative line profiles extracted at different lateral positions (Figure 4). The measurements revealed that the corrosion-induced chemical response was not spatially uniform across either alloy surface. Instead, both pH and H_2_ exhibited localized regions with elevated signals, demonstrating the development of spatially heterogeneous corrosion activity. The heterogeneity was particularly pronounced for 0Gd. Distinct local maxima of both pH and H_2_ were observed within the mapped area. The local H_2_ concentration reached approximately 229 μmol L^−1^ at the most active position, while the local pH increased to approximately 9.1. The corresponding line profiles further confirmed that these signals were spatially confined rather than uniformly distributed across the surface. Such localized enrichment indicates that the cathodic reaction was preferentially concentrated in specific regions of the alloy, producing local electrochemical “hot spots”.

In comparison, the 3Gd alloy exhibited a substantially weaker local response. The local H_2_ concentration remained much lower, with the highest measured value being approximately 76 μmol L^−1^, while the pH distribution remained closer to the bulk HBSS condition. Although spatial variations were still detectable, the corresponding gradients were less pronounced than those observed for 0Gd. The difference between the two alloys is significant because it demonstrates that Gd addition influences not only the average magnitude of the corrosion response but also its spatial organization. At the early to-intermediate stage of degradation, 0Gd more readily developed strongly active local regions characterized by simultaneous H_2_ accumulation and alkalization, whereas 3Gd exhibited a considerably less intense spatially heterogeneous response.

The difference between the two alloys is particularly evident when the local chemical maps are considered together with the surface morphology. At 2 h, the 0Gd surface exhibited pronounced local pH and H_2_ maxima in regions where filiform corrosion was observed, indicating that the development of filiform corrosion was accompanied by a strongly localized and chemically active interfacial microenvironment. In contrast, no obvious filiform corrosion was observed on the 3Gd surface within the mapped area, and its local pH and H_2_ distributions remained comparatively weak and spatially less pronounced. Thus, at this stage, the 0Gd and 3Gd samples represented two distinct interfacial corrosion states: a localized and highly active state associated with filiform corrosion for 0Gd, and a comparatively less localized state without obvious filiform corrosion for 3Gd. This spatially resolved comparison at 2 h raises an important question about how these distinct interfacial states develop with further immersion. To address this question, the mapping resolution was increased and the local pH and H_2_ distributions were subsequently monitored at multiple immersion times from 3 to 18 h.

**Figure 5:**
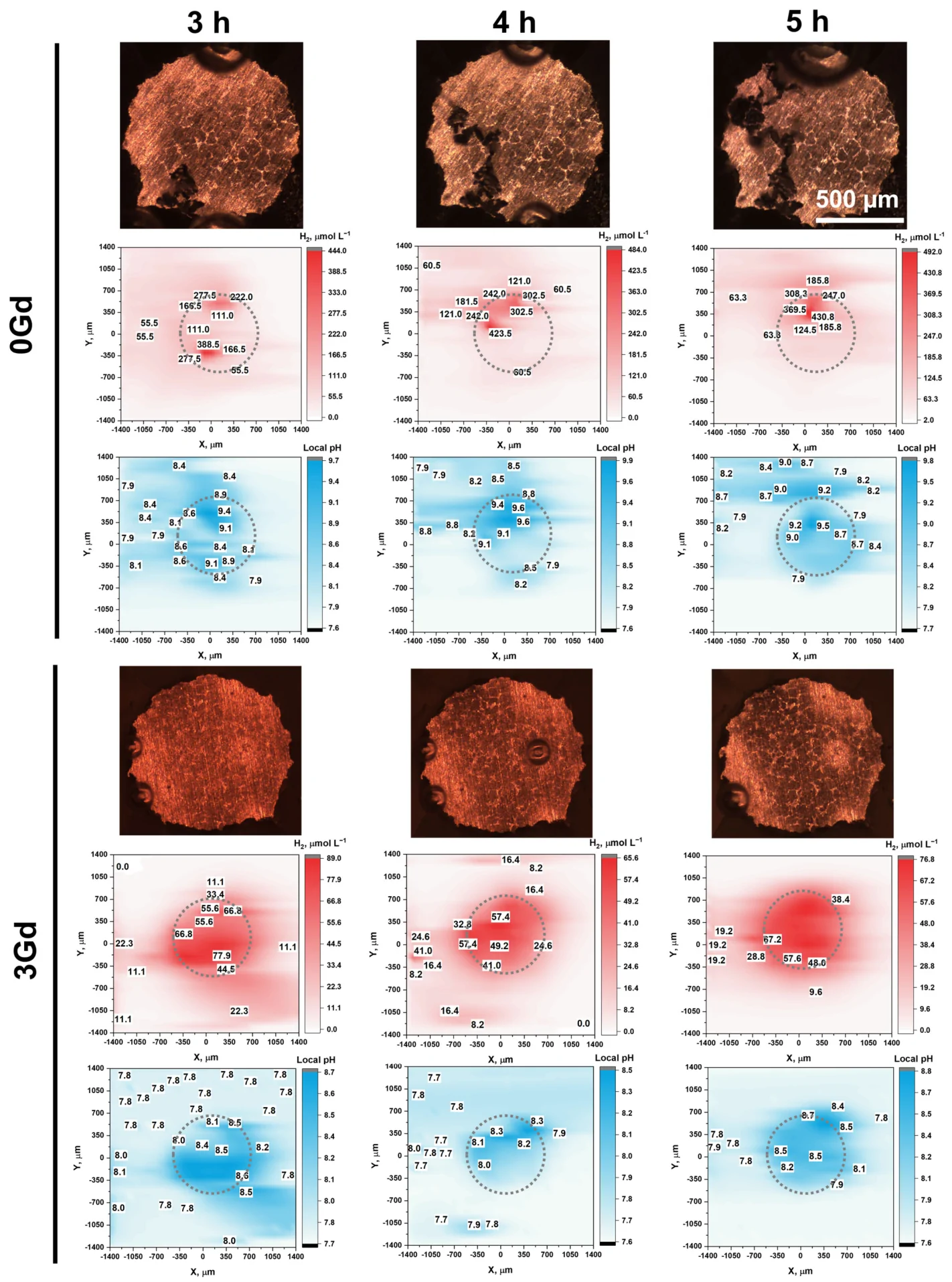
The evolution of the horizontal distribution of local pH and H_2_ level at above the surface of 0Gd and 3Gd exposed to HBSS from 3 to 5 h. The grey dash circles indicate the sample area.

The evolution of the local H_2_ and pH distributions from 3 to 5 h is shown in Figure 5. This period represents a critical stage during which the difference between 0Gd and 3Gd becomes increasingly pronounced. For 0Gd, the interfacial H_2_ distribution progressively intensified with increasing immersion time. At 3 h, the maximum local H_2_ concentration was approximately 388.5 μmol L^−1^. This value further increased to approximately 423.5 μmol L^−1^ at 4 h and approximately 430.8 μmol L^−1^ at 5 h. At the same time, the corresponding pH maps revealed the development of increasingly pronounced alkaline regions. The elevated local pH and H_2_ level became concentrated within specific areas of the surface.

The progressive increase in the amplitude and spatial concentration of the H_2_ signal suggests that corrosion activity on 0Gd became increasingly localized during this period. Certain regions developed into increasingly active sites where cathodic reactions and the associated accumulation of H_2_ and OH^−^ were strongly enhanced. In contrast, the 3Gd alloy maintained a considerably lower local H_2_ level throughout the same period. The maximum local H_2_ concentrations remained at approximately 60–80 μmol L^−1^, while the corresponding local pH distributions generally remained within a relatively moderate alkaline range. The spatial maps therefore showed much less pronounced H_2_ and pH hot spots than those observed for 0Gd. Although the high-resolution mapping required approximately 1 h for each complete scan, this acquisition strategy was intentionally adopted to prioritize spatial resolution at the selected immersion stages. The corrosion process had developed beyond the initial rapid transient response, allowing a longer acquisition period to be used to obtain substantially denser spatial information. In this regard, the longer acquisition time represents a deliberate balance between temporal and spatial resolution for local pH and H_2_ distributions. The same measurement configuration and data-processing procedure were maintained throughout the mapping experiments to enable comparison between different immersion stages.

**Figure 6:**
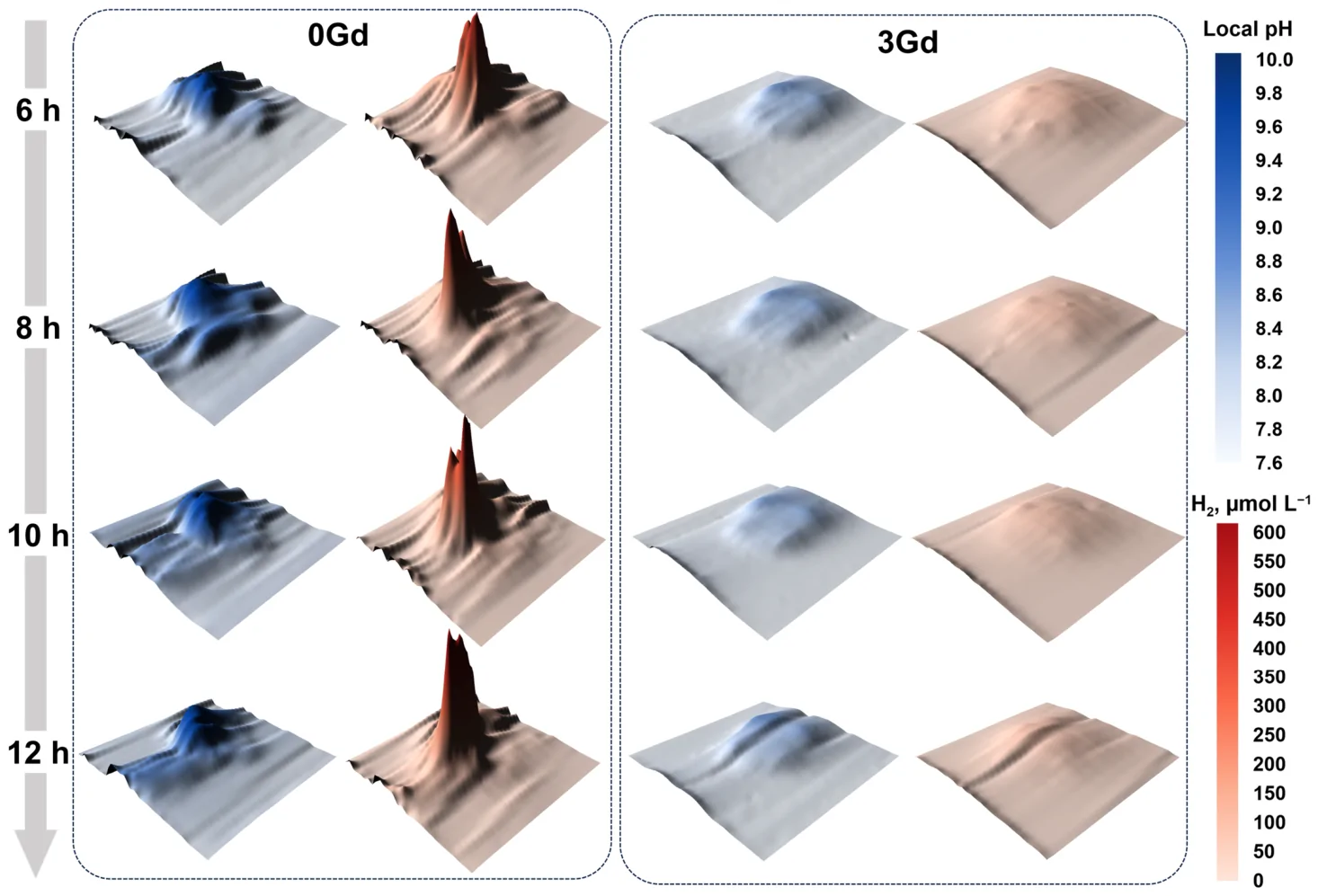
The evolution of the horizontal distribution of local pH and H_2_ level at above the surface of 0Gd and 3Gd exposed to HBSS from 6 to 12 h.

These differences of both local pH and H_2_ level become even clearer between 0Gd and 3Gd when the degradation process is extended to 6–12 h. As shown in Figure 6, both local pH and H_2_ distributions were reconstructed as three-dimensional surfaces to visualize the temporal evolution of the corrosion microenvironment.

For 0Gd, the three-dimensional maps demonstrate a progressive sharpening of the local pH and H_2_ peaks from 6 to 12 h. The local H_2_ signals in particular developed increasingly prominent peaks, indicating that the cathodic activity became progressively concentrated within specific surface regions. The corresponding pH distributions also exhibited localized alkaline peaks. Thus, the spatial contrast between active and relatively inactive regions became increasingly evident with immersion time. Such evolution suggests a transition from initially distributed corrosion activity toward increasingly localized corrosion behavior. Once a highly active region is established, the local electrochemical environment generated around that region may further influence the subsequent degradation process. Local alkalization, H_2_ accumulation, changes in surface coverage, and the development or disruption of corrosion products can all modify the local reaction conditions. The present data demonstrate the resulting chemical consequence, namely the formation of increasingly pronounced pH and H_2_ hot spots. In contrast, the 3Gd alloy maintained much flatter local pH and H_2_ landscapes throughout the 6–12 h period. Although local variations were still present, the corresponding peaks were considerably broader and less pronounced than those of 0Gd. This indicates that the corrosion activity remained comparatively distributed and less concentrated in highly active local sites. Taken together, Figure 5 and Figure 6 demonstrate that the 0Gd surface progressively evolves toward a strongly heterogeneous state characterized by intense local H_2_ accumulation and alkalization, whereas 3Gd maintains a relatively subdued and spatially homogeneous microenvironment over the same period.

**Figure 7:**
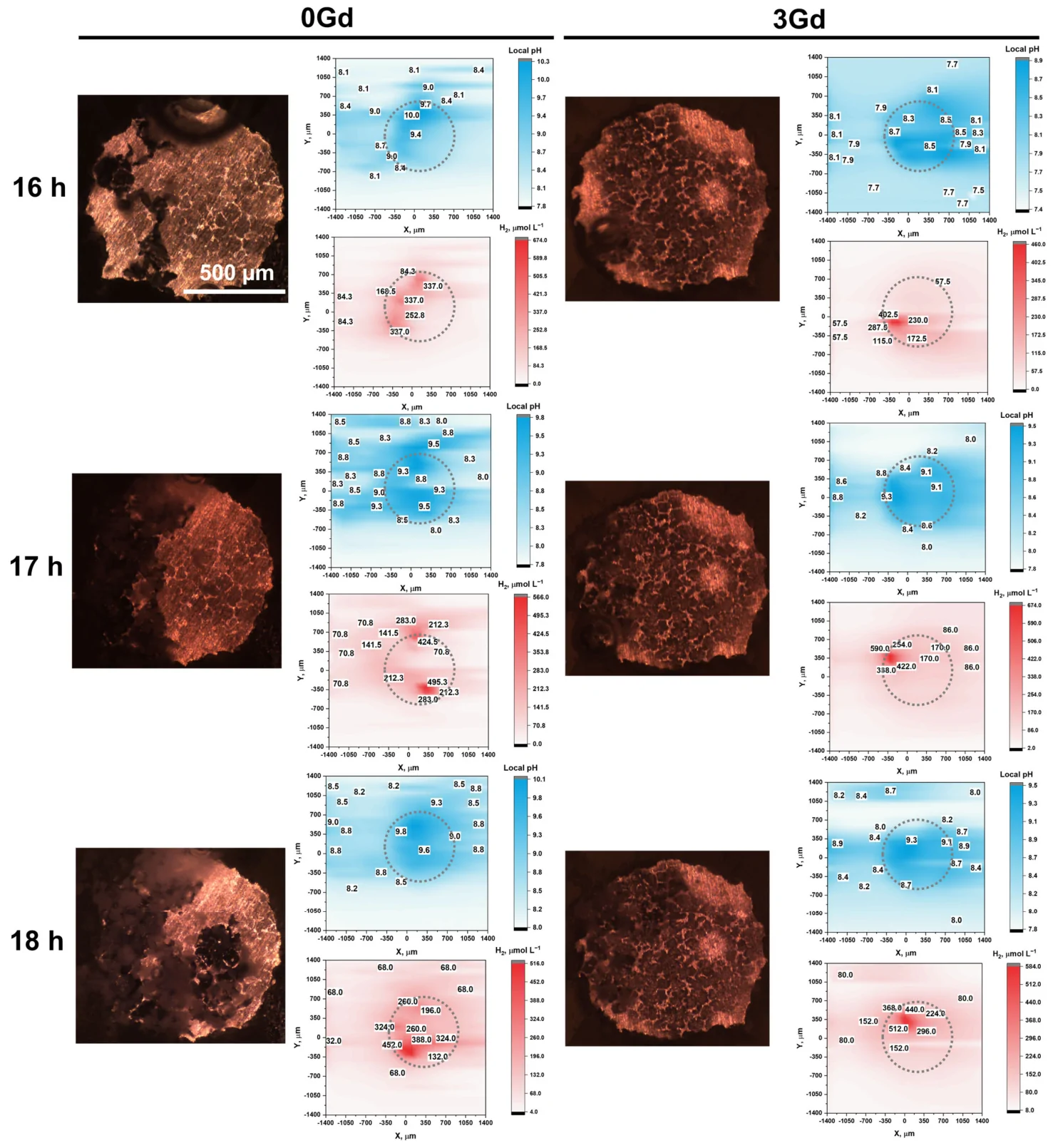
The evolution of the horizontal distribution of local pH and H_2_ level at above the surface of 0Gd and 3Gd exposed to HBSS from 16 to 18 h. The grey dash circles indicate the sample area.

The further evolution of the local pH and H_2_ distributions at 16, 17, and 18 h is presented in Figure 7. At this stage, the difference between the two alloys becomes more complex than that observed during the early and intermediate stages. For 0Gd, pronounced local alkaline regions were still present after prolonged immersion. At 16 h, the maximum local pH reached approximately 10.0, accompanied by a local H_2_ concentration of approximately 337 μmol L^−1^. At 17 h, the maximum H_2_ concentration increased further to approximately 495 μmol L^−1^, while the maximum local pH remained around 9.5. At 18 h, the maximum H_2_ concentration remained high, reaching approximately 452 μmol L^−1^, with a local pH of approximately 9.8. These results indicate that the highly active local corrosion sites developed during the earlier stages remained chemically active during prolonged degradation.

Interestingly, the 3Gd alloy exhibited a different late-stage response. Although the local pH remained substantially lower than that of 0Gd, local H_2_ concentration increased considerably at selected local positions. At 16 h, the maximum local H_2_ concentration reached approximately 402.5 μmol L^−1^, increasing to approximately 590 μmol L^−1^ at 17 h and remaining high at approximately 512 μmol L^−1^ at 18 h. In contrast, the corresponding local pH values were generally within approximately 8–9.3. Thus, 3Gd could still develop regions with intense H_2_ accumulation during prolonged immersion. Although H_2_ evolution and OH^−^ generation originate from related cathodic reactions, the measured local pH is additionally influenced by OH^−^ transport, diffusion, buffering reactions in HBSS, surface reactions, and changes in the corrosion products layer, which act as a dynamically evolving interfacial barrier.

Although the relationship between the Gd distribution observed by SEM/EDS and the local pH/H_2_ hotspots measured by microsensors is not necessarily a direct one-to-one correspondence, the consistent difference in spatial organization between the 0Gd and 3Gd alloys supports a microstructure-associated origin of the heterogeneous corrosion microenvironment. Also, the statistical uncertainties such as standard deviations were not calculated for the spatial maps. However, the major corrosion trends observed here, including the distinct localized corrosion response of the 0Gd and 3Gd alloys and the influence of Gd addition on corrosion behavior, are consistent with our previous systematic investigation of the same Mg-Y-Al-Zn-Gd alloy system in a NaCl electrolyte [53]. The calibration procedures described above were applied to quantitatively convert the microsensor signals, while the agreement with the previously reported alloy-dependent corrosion behavior provides additional support for the consistency of the present observations. Therefore, simultaneous measurement of local pH and H_2_ offers information that cannot be obtained from either parameter alone. H_2_ provides a direct indicator of intense local cathodic activity, whereas local pH reflects the net chemical consequence of OH^−^ generation and its subsequent transport and consumption. The detected H_2_ level reflects the combined effects of local H_2_ generation and subsequent transport processes, including diffusion, convection, dissolution into the electrolyte, and possible formation and detachment of H_2_ bubbles at the Mg surface [29,54,55]. Their combined spatial distribution can consequently distinguish different modes of interfacial corrosion activity. The spatial distribution and temporal evolution of the local reaction environment provide critical information about how corrosion activity develops and becomes localized. The combined local pH and H_2_ mapping used in this study therefore provides a direct approach for resolving the dynamic interfacial microenvironment of degrading Mg alloys.

## 3. Materials and Methods

### 3.1. Materials and Sample Preparation

The Mg alloys investigated in this study were Mg-6.7Y-1YAl-2.5Zn and Mg-6.7Y-1YAl-2.5Zn-3Gd, respectively denoted as 0Gd and 3Gd. The detailed preparation, elemental composition, microstructure characterization, and corrosion properties of these Mg alloys in a NaCl electrolyte are described in [53]. The 3 wt.% Gd composition was selected as a representative Gd-containing alloy with distinguishable microstructural and corrosion characteristics from the Gd-free alloy [53], investigating how different corrosion behaviors are reflected in the spatiotemporal evolution of the local pH and H_2_ microenvironment. The alloy specimens were embedded into the epoxy resin using cold-mounting procedures, exposing a circular working surface with a diameter of 1 mm. The specimens were consecutively ground using SiC papers from 1200 grit to 3000 grit and cleaned in absolute ethanol and deionized water. Commercial Hanks’ balanced salt solution (HBSS, BL562A, Biosharp, China) was used as the electrolyte throughout the experiments.

### 3.2. Microstructural Characterization

The initial surface morphology and elemental distribution of the 0Gd and 3Gd alloys were characterized using scanning electron microscopy (SEM, Sirion 200, FEI from Eindhoven, The Netherlands). The specimens were underwent mechanical polishing using a 2.5 μm diamond suspension and final etching with 4% nitric acid in ethanol prior to SEM characterization. The SEM images were used to compare the surface morphology and microstructural features of the two alloys, while the corresponding EDS maps were used to determine the spatial distribution of the major alloying elements and to confirm the presence and distribution of Gd in the 3Gd alloy.

### 3.3. Local pH and H_2_ Microsensor Measurements

Spatially resolved measurements of local pH and H_2_ concentration above the alloy surface were performed using a glass-type potentiometric pH microsensor (pH-25, Unisense™, Aarhus, Denmark) and a Clark-type amperometric H_2_ microsensor (H2-25, Unisense™, Denmark), connected to a Microsensor Multimeter (Unisense™, Denmark) integrated with a motion system (Applicable Electronics™, Sandwich, MA, USA). The tip diameter of both microsensors is around 25 μm. A mini Ag/AgCl reference electrode was connected to the pH microsensor. Prior to measurements, the pH microsensor was calibrated using commercial pH buffer solutions. The H_2_ microsensor was calibrated using a two-point method: a zero point in HBSS and a second point in the same electrolyte saturated with a 5% H_2_/95% Ar gas mixture, considering the salinity of HBSS. Using the same electrolyte composition for H_2_ calibration minimizes potential deviations associated with the electrolyte matrix and salinity. The distance between microelectrodes was kept at 50 µm in horizontal planes via micromanipulators under microscopic observation. The sensor position was controlled using a motorized three-dimensional positioning system, allowing the probe to be accurately moved in the vertical (Z) and horizontal (X–Y) directions. The measurement height of 100 μm was selected to probe the near-interface region where diffusion-controlled concentration gradients are expected to develop during degradation, while avoiding physical collision between the probe and the evolving surface. The HBSS electrolyte in this small cell was maintained under the hydrodynamic condition with a flow rate of 1.0 mL min^−1^ controlled by a peristaltic pump (LabVII, Shenchen, China). The volume of the cell for local measurements was 5 mL, which was fully renewed in every 5 min and the used electrolyte was not recirculated. This helps to stabilize the bulk pH while still allowing the evolution of local pH gradients near the degrading Mg specimen. A series of sequential spatiotemporal measurements, including time-resolved monitoring, vertical profiling, and spatial mapping, were performed to characterize the evolution of the interfacial corrosion microenvironment, following the spatially resolved microsensor measurement strategy established in our previous studies [16,29,34]. The measurements were conducted progressively from a fixed position to the vertical direction and finally to the lateral plane, enabling the temporal evolution, concentration gradients, and spatial heterogeneity of local pH and H_2_ to be evaluated.

### 3.4. Experimental Procedures for Spatially Resolved Local Measurements

#### 3.4.1. Immediate Time-Resolved Measurements Above the Surface Center

To characterize the immediate response of the alloy/electrolyte interface after immersion, the local pH and H_2_ concentrations were monitored continuously above the center of the alloy surface during the first 5 min of exposure to HBSS. After the alloy specimen was introduced into the HBSS-containing cell, the microsensor was positioned above the central region of the surface at a fixed vertical distance. The local pH and H_2_ signals were then recorded continuously as a function of immersion time with the optical image of the alloy surface simultaneously captured. This measurement mode was designed specifically to resolve the earliest stage of corrosion initiation.

#### 3.4.2. Vertical Profiling from the Alloy Surface to the Bulk Electrolyte

To determine the spatial extent of the corrosive microenvironment, vertical profiles of local pH and H_2_ were measured along the direction normal to the alloy surface. The microsensors were initially positioned either close to the alloy surface or in the bulk electrolyte, and were subsequently moved stepwise along the vertical direction. Two scanning directions were employed: from the alloy surface toward the bulk electrolyte and from the bulk electrolyte toward the alloy surface. During each scan, both local pH and H_2_ microsensors were moving between 100 μm and 2000 μm above the specimen midpoint at successive vertical positions, generating a concentration profile as a function of the distance from the alloy surface. The purpose of this measurement was to distinguish the interfacial response from changes in the bulk electrolyte.

#### 3.4.3. Horizontal Mapping of the Interfacial Microenvironment

Horizontal distributions of local pH and H_2_ were obtained by scanning the microsensors laterally across the alloy surface at a fixed vertical distance above the surface. The measurement region was defined according to the visible specimen area under the optical microscope. A large-area horizontal mapping was continuously performed over a sample-centered area of 2800 μm × 2800 μm, monitoring the local progression above the Mg substrate in HBSS. This field of view was deliberately chosen to cover both the sample surface region and the surrounding electrolyte region outside the sample boundary (sample diameter: 1 mm). The regions scanned above the embedding resin and bulk electrolyte were used as an internal spatial comparison with the interfacial region above the degrading Mg. This configuration enables direct in situ comparison between the material-influenced region and the adjacent bulk-like solution within the same measurement frame, thereby providing an internal spatial reference (control region) for evaluating pH and H_2_ variations induced by the specimen.

The horizontal mapping experiment was first performed after 2 h of immersion to characterize the early spatial distribution of corrosion activity. A relatively large step size of 200 μm was first selected to balance spatial coverage and measurement duration. The mapping consisted of a 15 × 15 grid, corresponding to 225 measurement points. At each position, the local pH or H_2_ concentration was recorded with an average acquisition time of approximately 3 s per point, resulting in a total mapping time of approximately 15 min for each scan considering the probe movements. This relatively coarse but rapid mapping strategy was designed to capture the overall spatial distribution and heterogeneity of the corrosion-induced pH and H_2_ responses over the entire sample surface during the early stage of degradation. The collected data points were subsequently interpolated to generate two-dimensional distribution maps. Representative horizontal line profiles were also extracted from the maps to quantitatively compare the spatial variation in local pH and H_2_ across selected regions of the surface.

To further resolve the temporal evolution of the spatially heterogeneous corrosion microenvironment, higher-resolution horizontal mapping was performed at selected immersion times following the initial large-area survey. A smaller scanning step size of 100 μm was employed to improve the spatial resolution of the local pH and H_2_ distributions. The same probe-to-surface configuration and scanning procedure were maintained throughout the measurements, while the reduced step size resulted in a longer acquisition time of approximately 1 h for each complete map. Continuous measurements were performed at selected immersion times between 3 and 18 h, and the resulting spatially resolved datasets were used to track the evolution, persistence, and redistribution of local pH and H_2_ signals during degradation. This approach provided a balance between spatial resolution and temporal coverage, enabling the progressive changes in the interfacial corrosion microenvironment to be characterized over an extended immersion period.

### 3.5. Data Processing and Visualization

The local pH and H_2_ signals were converted into quantitative values using the calibration relationships established for the corresponding microsensors. For vertical measurements, the local pH and H_2_ values were plotted as a function of the distance from the alloy surface. For horizontal measurements, the measured values were assigned to their corresponding spatial coordinates and interpolated to generate two-dimensional maps of the local pH and H_2_ distributions. Representative line profiles were extracted from the spatial maps for quantitative comparison of local variations between 0Gd and 3Gd. The same data-processing and visualization procedures were applied to both alloys.

## 4. Conclusions

In this study, spatially resolved local pH and H_2_ measurements were employed to directly visualize the evolution of the interfacial corrosion microenvironment during degradation of 0Gd and 3Gd Mg alloys in HBSS. The main conclusions can be summarized as follows:

1. Both local pH and H_2_ concentration rapidly increased at the interfaces of 0Gd and 3Gd immediately after immersion in HBSS. The corrosion-induced changes were strongly localized near the Mg surface, resulting in pronounced vertical gradients between the interface and bulk electrolyte.
2. The two alloys exhibited different spatial evolutions of the localized corrosion microenvironment during immersion. At the initial stage, local pH and H_2_ level for 0Gd were even slightly lower than those of 3Gd. At 2 h, however, 0Gd exhibited pronounced local pH and H_2_ hot spots associated with the occurrence of filiform corrosion. From 6 to 12 h, these localized features on 0Gd became progressively more pronounced, whereas 3Gd maintained a comparatively weaker and more spatially distributed response.
3. With prolonged immersion, localized H_2_ accumulation could also develop on 3Gd, while the corresponding local alkalization remained relatively moderate. This further demonstrates that Mg corrosion evolves dynamically in both space and time, and that local H_2_ evolution and pH provide complementary information for characterizing the interfacial corrosion microenvironment.

Overall, these findings demonstrate that Mg degradation in HBSS is a dynamic and heterogeneous process. Simultaneous mapping of local pH and H_2_ provides a powerful approach for resolving this evolving interfacial microenvironment and offers a comprehensive perspective on the spatiotemporal evolution of localized corrosion in Mg alloys.

## Data Availability

The original contributions presented in this study are included in the article. Further inquiries can be directed to the corresponding authors.
